# Case of Mostoiditis Complicating Purulent Otitis Media Cured by Enlarging the Drum; Perforation and Syringing the Tympanitic Cavity*Read by title at the meeting of the Western Ophthalmological, Otological, Laryngological and Rhinological Association, April, 1897.

**Published:** 1897-06

**Authors:** W. S. Scheppegrell

**Affiliations:** Vice-President of the American Laryngological, Rhinological and Otological Society, etc., New Orleans, La.


### TEXAS MEDICAL JOURNAL.

ESTABLISHED JULY, 1885.

PUBLISHED MONTHLY.—SUBSCRIPTION $2.00 A YEAR.

VOL. XII. AUSTIN, JUNE, 1897. NO. 12.

### Original Contributions.

For the Texas Medical Journal.

### Case of Mastoiditis Complicating Purulent Otitis Media Cured by Enlarging the Drum; Perforation and Syringing the Tympanitic Cavity.*

BY W. SCHEPPEGRELL, A. M., M. D.,
Vice-President of the American Laryngological, Rhinological and Otological Society, etc., New Orleans, La.

IN MARCH 14, 1895, Dr. McLawrence, of Monroe, La., called at my office and requested my advice in a case of acute mastoiditis. The history of the case was as follows: The patient, a railroad officer, 40 years of age, affected with rhinitis atrophica, had developed an acute purulent otitis media about two months ago. This case had progressed favorably under treatment; the discharge had almost disappeared, and the perforation on the drum had become reduced to a small orifice. A week before the patient was called to my attention, however, he had been overtaken by a rain storm, which, immediately aggravated all the symptoms of the case. The discharge became more abundant and more purulent in character, and the temperature reached 103 degrees. An inflammatory swelling of the mastoid now developed, and to such an extent that it projected the auricle forward, which now assumed the appearance character-

* Read by title at the meeting of the Western Ophthalmological, Otological, Laryngological and Rhinological Association, April, 1897.

istic of aggravated cases of mastoiditis. The patient was depressed, dull and listless.

When I examined the case, I found the ear and the adjoining region in the condition already described. The mastoid was swollen, inflamed, and painful on pressure; the temperature was 101 degrees, but had been much higher during the day.

This case presented all the usual symptoms demanding an immediate operation, and it was then decided that if the symptoms did not show marked improvement within twenty-four hours, the opening of the mastoid cells and the antrum would at once be undertaken. In order to give the patient the benefit of free drainage and disinfection of the tympanic cavity, I then made a V shaped incision in the anterior posterior part of the drum, using the small perforation as the apex of the angle. A small tympanic canula was then passed through the orifice and the cavity gently but thoroughly syringed with a ten per cent. solution of peroxide of hydrogen, this procedure being repeated the same evening. The following day the appearance of the patient had improved in a marked manner; the depression and dullness had disappeared, the temperature was 99.3 degrees, and the mastoid process, although still swollen, was not so painful to the touch. Under this treatment the condition of the patient rapidly improved, and five days later the case was again placed in Dr. McLawrence's hands, who afterwards reported to me the full recovery of the patient.

This case is reported not only on account of its clinical history, but also as demonstrating the fact that conservative methods may sometimes enable us to avoid more dangerous procedures in cases in which the indications are very pronounced. This conservatism, however, should not be carried to an extreme, and the case should be carefully watched so that prompt steps may at once be taken to more radical methods.

I will also give you a short clinical history of another case in which an operation on the mastoid was avoided, and the patient made a full recovery. This case, a patient of 47 years, was referred to me by Dr. John B. Elliott, of New Orleans. It was at first a case of purulent otitis media, the result of grippe, which afterwards developed inflammation of the mastoid cells. In this case there was light inflammatory swelling of the mastoid region, but not very pronounced. The post-auricular region was very tender to pressure, and the pain in the mastoid region was of such an excruciating character that it could be quieted only by means of large doses of morphine.

The indication in this case for opening the mastoid cells was the agonizing character of the pain which persisted for weeks. The patient, however, had suffered for several years from an affection of the heart, so that chloroform or ether narcosis could be given only as an extreme resort. An expective treatment was, therefore, followed out by thorough disinfection of the tympanic cavities, counter-irritants, etc. The paroxysms of pain continued daily, and could be quieted only by opiates, and did not disappear for over two months. They then subsided, and the patient has made a full recovery.

From the study of the clinical history of this case, I do not recommend that, on account of the ultimate result in this case, a similar treatment be advocated in other cases, unless the contra-indications to a radical operation are as pronounced. The thorough drainage of the antrum and cells in this case would have avoided the depressing effects of pain continuing for such a long period of time, and also the effects of narcotics, which the relief of the pain necessitated.

